# Supplementary material for: High-Efficiency and Broadband Near-Infrared Bi-Functional Metasurface Based on Rotary Different-Size Silicon Nanobricks
Source: Nanomaterials (Basel). 2019 Dec 7;9(12):1744. doi: 10.3390/nano9121744 (PMC6955931; doi:10.3390/nano9121744)
Supplement: Supplementary file 1 [file nanomaterials-09-01744-s001.pdf]

nanomaterials

MDPI

Supplementary Information for:

High-Efficiency and Broadband Near-Infrared Bi-Functional Metasurface Based on Rotary Different-Size Silicon Nanobricks

Wei Wang [1, 2], Chong Guo [1], Jingluo Tang [1], Zehan Zhao [1], Jicheng Wang[3], Jinghua Sun [2], Fei Shen[2], Kai Guo[4], and Zhongyi Guo[2] [4,*]

[1] Department of Mathematics and Physics, Shijiazhuang Tiedao University, Shijiazhuang 050043, China; wangwg@stdu.edu.cn (W.W.); guocong122@163.com (C.G.); 1640392924@qq.com (J.T.); xvxvxiaozhao@163.com (Z.Z.);
[2] School of Electrical Engineering and Intelligentization, Dongguan University of Technology, Dongguan 523808, China; sunjh@dgut.edu.cn (J.S.); shenfei@hfut.edu.cn (F.S.);
[3] School of Science, Jiangnan University, Wuxi 214122, China; jcwang@jiangnan.edu.cn (J.W.);
[4] School of Computer and Information, Hefei University of Technology, Hefei 230009, China; kai.guo@hfut.edu.cn (K.G.)
* Correspondence: guozhongyi@hfut.edu.cn; Tel.: +86-18655151981

Supplementary Note 1: Broadband characteristics of bi-functional beam deflector

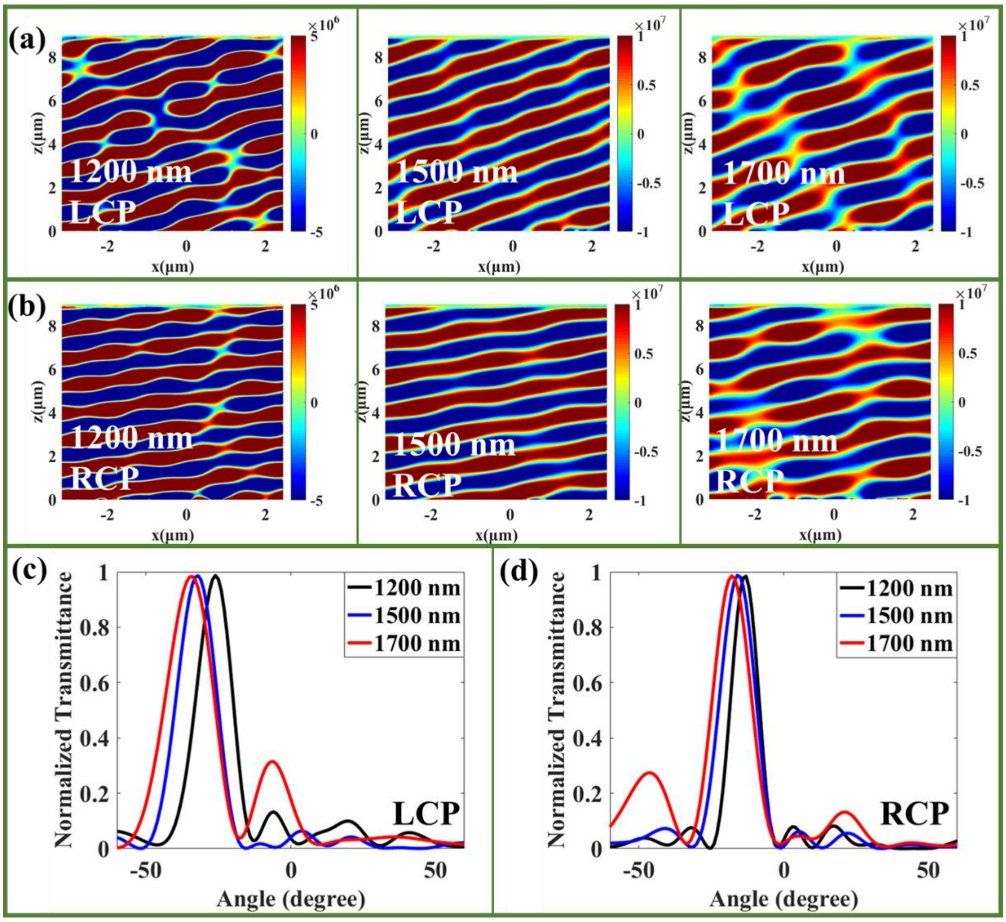

Figure S1. Electric field distributions of the bi-functional beam deflector under LCP (a) and RCP (b) incidences at the incident wavelengths of 1200, 1500, and 1700 nm respectively (The color scale units of the electric field are V/m.). The transmittances as a function of scattered angles under LCP (c) and RCP (d) incidences with different wavelengths.

Supplementary Note 2: Broadband characteristics of bi-functional metalens

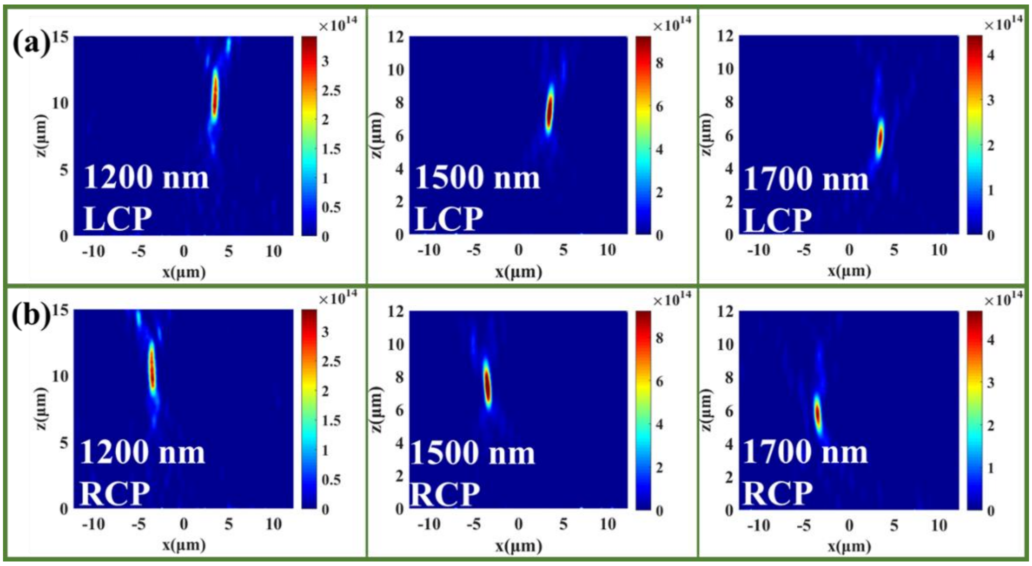

Figure S2. Intensity distributions of the bi-functional metalens under LCP (a) and RCP (b) incidences at the incident wavelengths of 1200, 1500, and 1700 nm respectively (The color scale units of the intensity are $(V/m)^2$).

Supplementary Note 3: Broadband characteristics of BVPP.

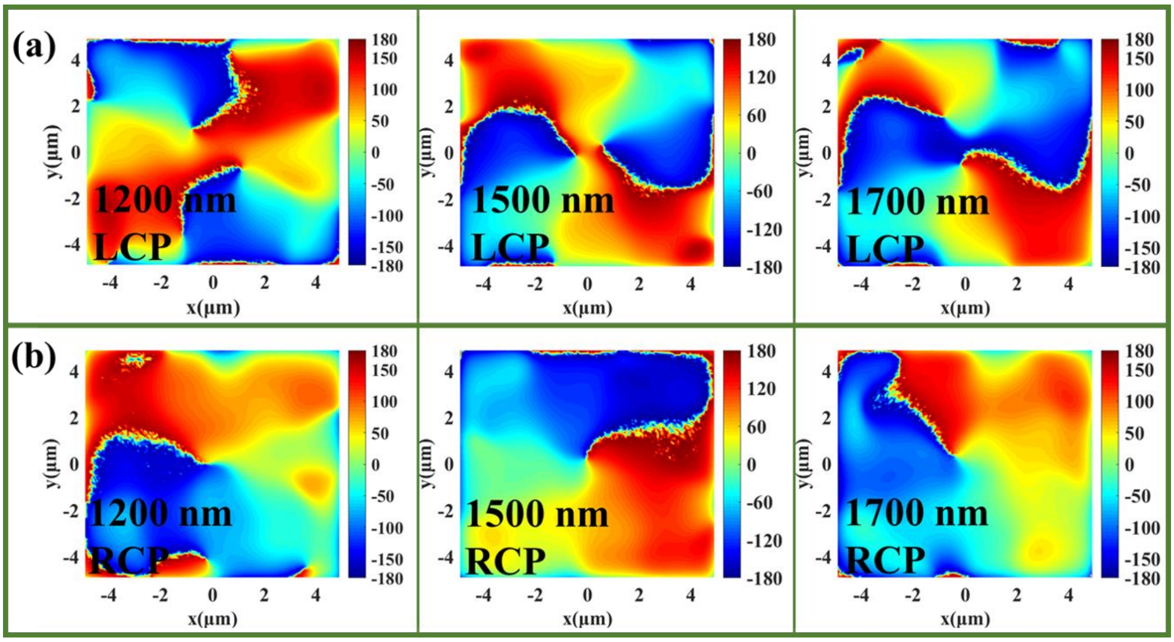

Figure S3. The phase distributions of the produced vortex beam under LCP (a) and RCP (b) incidences at the incident wavelengths of 1200, 1500, and 1700 nm respectively (The color scale unit of the phase is deg).

Supplementary Note 4: Broadband characteristics of one unit cell.

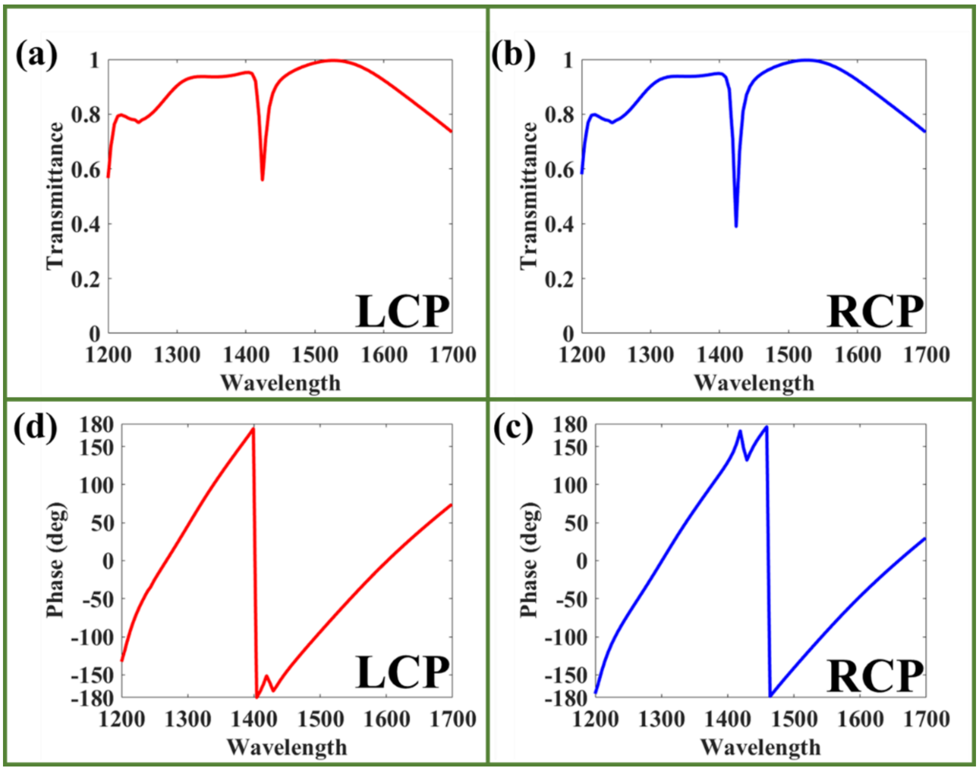

Figure S4. The transmittance (a) and phase (d) of transmitted light through the unit cells (L=230 nm, W=390 nm, θ =11.3°) for LCP incidence in the incident wavelength range of 1200-1700 nm. The transmittance (b) and phase (c) of transmitted light through the unit cells (L=230 nm, W=390 nm, θ =11.3°) for RCP incidence at the incident wavelength range of 1200-1700 nm.
